# Inhibition of Rho-Kinase Downregulates Th17 Cells and Ameliorates Hepatic Fibrosis by *Schistosoma japonicum* Infection

**DOI:** 10.3390/cells8101262

**Published:** 2019-10-16

**Authors:** Wei Zhou, Yingying Yang, Congjin Mei, Panpan Dong, Shasha Mu, Hongchu Wu, Yonghua Zhou, Yi Zheng, Fukun Guo, Jun-Qi Yang

**Affiliations:** 1 Key Laboratory of National Health Commission on Parasitic Disease Control and Prevention, Jiangsu Provincial Key Laboratory on Parasite and Vector Control, Jiangsu Institute of Parasitic Diseases and Public Health Research Center of Jiangnan University, Wuxi 214064, Jiangsu, China; zhouweijipd@163.com (W.Z.); yvonne_0713@163.com (Y.Y.); meicongjin@163.com (C.M.); mufen1989@163.com (P.D.); mss2013@126.com (S.M.); whcjipd@163.com (H.W.); toxo2001@163.com (Y.Z.); 2 Division of Experimental Hematology and Cancer Biology, Cincinnati Children’s Hospital Medical Center, Cincinnati, OH 45229, USA; yi.zheng@cchmc.org

**Keywords:** RhoA-ROCK, fasudil, Th17, *Schistosoma japonicum*, hepatic fibrosis

## Abstract

Background: Schistosomiasis is an immunopathogenic disease in which Th17 cells play vital roles. Hepatic granuloma formation and subsequent fibrosis are its main pathologic manifestations and the leading causes of hepatic cirrhosis, and effective therapeutic interventions are lacking. In this study, we explored the effects of fasudil, a selective RhoA–Rho-associated kinase (ROCK) inhibitor, on Th17 cells and the pathogenesis of schistosomiasis. Methods: Mice were infected with *Schistosoma japonicum* and treated with fasudil. The worm burden, hepatic granuloma formation, and fibrosis were evaluated. The roles of fasudil on Th17, Treg, and hepatic stellate cells were analyzed. Results: Fasudil therapy markedly reduced the granuloma size and collagen deposit in livers from mice infected with *S. japonicum*. However, fasudil therapy did not affect the worm burden in infected mice. The underlying cellular and molecular mechanisms were investigated. Fasudil suppressed the activation and induced the apoptosis of CD4^+^ T cells. Fasudil inhibited the differentiation and effector cytokine secretion of Th17 cells, whereas it upregulated Treg cells in vitro. It also restrained the in vivo interleukin (IL)-4 and IL-17 levels in infected mice. Fasudil directly induced the apoptosis of hepatic stellate cells and downregulated the expressions of hepatic fibrogenic genes, such as *collagen type I* (*Col-I*), *Col-III*, and *transforming growth factor-1* (*TGF-β1*). These effects may contribute to its anti-pathogenic roles in schistosomiasis. Conclusions: Fasudil inhibits hepatic granuloma formation and fibrosis with downregulation of Th17 cells. Fasudil might serve as a novel therapeutic agent for hepatic fibrosis due to schistosome infections and perhaps other disorders.

## 1. Introduction

Schistosomiasis remains a severe public health problem in the endemic areas in many developing countries [1,2]. It is an immunopathogenic disorder caused by blood flukes from the genus *Schistosoma*. There are three main species pathogenic to human: *Schistosoma japonicum* (*S. japonicum*), *S. mansoni*, and *S. haematobium.* When schistosome eggs are trapped in the venules, delayed-type hypersensitivity reactions are evoked and lead to the formation of circumoval granulomas and subsequent fibrosis in the affected tissues, such as the liver and intestines [3,4]. This is the main pathologic manifestation of schistosomiasis and the leading cause of hepatic cirrhosis in the late stage. To date, the pathogenesis for granuloma formation and fibrosis remains elusive, and effective therapeutic interventions are lacking [2,5]. 

Regarding the pathogenesis of schistosome infections, it is generally thought that T helper 1 (Th1) responses are elicited during the early phase by larval worms, succeeded by Th2 responses by deposited eggs. Egg deposit in the tissues is a determining factor driving Th2 responses in *S. mansoni* and *S. japonicum* infections in mice [4,6]. Recent studies have indicated that Th17 and regulatory T (Treg) cells are crucial in the pathogenesis of schistosome infections [7,8,9,10]. Hepatic fibrosis is mainly caused by liver-injury-mediated inflammation and activation of hepatic stellate cells (HSCs) [5,11]. The expressions of both interleukin (IL)-17 and its receptor IL-17RA are upregulated in hepatic fibrosis; IL-17 directly induces the production of collagen type I (Col-I) via signal transducer and activator of transcription 3 (Stat3) pathways in murine HSCs [12]. We recently found that IL-17 in the circulating blood is elevated from the early phase (3 weeks), peaked at 7 weeks, and was still maintained at a high level at 10–12 weeks post *S. japonicum* infection in mice [10]. These data indicate that Th17 cells may actively participate in the early anti-infection immunity and late immunopathogenesis for granuloma formation and fibrosis. 

The Rho GTPases belong to the Ras superfamily of small guanosine triphosphate (GTP)-binding proteins. They are nucleotide-dependent proteins behaving like molecular switches that cycle between an inactive, guanosine diphosphate (GDP)-bound, and an active, GTP-bound, state [13]. Among over 20 members of the family, three have been extensively studied: RhoA, CDC42, and Rac1. Rho GTPases are intracellular signaling proteins regulating multiple pathways involved in various important biological functions, such as cellular membrane transport, migration, differentiation, proliferation, and apoptosis. They play important regulatory roles in infection, inflammation, and oncogenesis, and become novel therapeutic targets [14,15,16,17]. 

Rho-associated kinase (ROCK) is one of the main downstream signaling molecules of RhoA. Fasudil, a selective inhibitor of ROCK, has been applied in RhoA functional studies and as clinic therapies for several disorders. Fasudil has been approved for clinical therapies of cerebral vasospasm in Japan and China [18]. It is also applied in patients with pulmonary hypertension [19], diabetic patients with left ventricular diastolic dysfunction [20], etc. For RhoA functional research, fasudil has been applied for fibrosis therapy in several models [21,22,23]. Fasudil may also inhibit Th17 cells. Fasudil treatment was related to the downregulation of IL-17 mRNA expression from cardiac tissues in a murine model of myocarditis [24]. Y27632, another RhoA-ROCK inhibitor, can suppress ovalbumin (OVA)-induced murine asthma with reduced IL-17 mRNA expression in lungs [25]. It also reduces IL-17 and IL-21 secretion from cord blood T cells in lupus patients [26]. With the combined use of conditional RhoA-deficient mice and fasudil, we found that RhoA orchestrated glycolysis for Th2 differentiation and OVA-induced asthma [27]. Most recently, we reported that RhoA genetic deficiency and its specific inhibitor Y16 impaired Th17 cell differentiation and alleviated house dust-mite-triggered allergic airway inflammation [28]. However, whether and how fasudil impacts Th17 cells and the immunopathogenesis of schistosomiasis remain to be elucidated.

In the current study, we first evaluated the roles of the RhoA-ROCK pathway in *S. japonicum* infection and the pathogenesis by using its selective inhibitor fasudil in a murine infection model. We then explored the underlying cellular and molecular mechanisms through which fasudil affects the pathogenesis of schistosomiasis. We found that fasudil therapy is able to inhibit the hepatic granuloma formation and subsequent fibrosis. This inhibition is related to the suppression of Th2 as well as Th17 cells, and to the induction of Treg cells. It is also related to the direct apoptotic role of fasudil to hepatic stellate cells. Fasudil might serve as a potential novel therapeutic agent for hepatic fibrosis due to schistosome infection and perhaps other disorders.

## 2. Materials and Methods

### 2.1. Mice and Parasites

Female C57BL/6 mice (8–10 weeks) were purchased from the College of Veterinary Medicine, Yangzhou University, Yangzhou, China, and maintained under specific pathogen-free conditions at the Jiangsu Institute of Parasitic Disease (JIPD; Wuxi, China). This study was conducted following the guidelines for the care and use of laboratory animals of the Institutional Animal Care and Use Committee of JIPD. *Schistosoma japonicum* (Chinese mainland strain) was obtained from JIPD (Wuxi, China). Mice were percutaneously infected with 15 cercariae of *S. japonicum* and injected intraperitoneally (i.p.) with fasudil (Tianjin Hongri Pharmaceutical Co., Ltd., Tianjin, China) at 0–30 mg/kg body weight daily for a total of 5–8 weeks starting prior to infection or post-infection as indicated.

### 2.2. Worm Burden and Hepatic Fibrosis

Adult worms were collected through the perfusion of portal and mesenteric veins from infected mice and counted. For fecal egg counting, fresh feces were collected, weighed, and suspended in normal saline, then passed through a 200-mesh sieve. After 3 sedimentations, eggs in the pellets were counted and expressed as eggs per gram (EPG) of feces. A portion of the right lobe of the liver was weighed and digested in 10 mL 4% potassium hydroxide (KOH) overnight at 37 °C, the eggs were counted, and is expressed as numbers per gram of livers. Another portion of the right lobe of the liver was digested for the hydroxyproline assay with a hydroxyproline detection kit (Nanjing Jiancheng Bioengineering Research Institute Co., Ltd., Nanjing, China), following the manufacturer’s protocol. Hydroxyproline is expressed as µg per gram of livers by reference to a standard curve. 

### 2.3. Histopathology

The large left lobe of the liver was fixed with 4% paraformaldehyde in phosphate buffer saline (PBS) and processed for histological examination with hematoxylin and eosin (H&E) and Masson trichrome staining. The areas of granulomas containing a single egg were measured under an Olympus BX51 microscope (Olympus Co., Tokyo, Japan) with the JEDA801D Image Analyzing System (Jiangsu Jeda, Nanjing, China). For every mouse, 3–5 sections were read, 20 granulomas or more were measured, and the mean area was calculated.

### 2.4. T Cell Activation and Differentiation

Naïve T cells were activated with plate-bound anti-CD3 (10 µg/mL) plus soluble anti-CD28 (2 µg/mL, BD Bioscience, San Jose, CA, USA) for 2 days. For T cell differentiation, CD4^+^ T cells were differentiated into Th17 or iTreg cells as previously reported [27,29]. Briefly, CD4^+^ T cells were stimulated by anti-CD3/CD28 for 4 days with anti-IFN-γ and anti-IL-4 (both 10 µg/mL) in the presence of TGF-β1 (5 ng/mL, for Treg), or TGF-β1 plus IL-6 (10 ng/mL, for Th17) (all obtained from R&D Systems, Minneapolis, MN, USA). Cells were restimulated with phorbol 12-myristate 13-acetate (PMA, 25 ng/mL) plus ionomycin (500 ng/mL; Sigma, St. Louis, MO, USA) for 5 h with GolgiStop (BD Bioscience, San Jose, CA, USA) in the last 2 h for intracellular cytokine staining; or without GolgiStop for cytokine assays in the culture supernatants by enzyme-linked immunosorbent assay (ELISA). Where indicated, fasudil (Selleck Chemicals, Houston, TX, USA) was added to the cultures.

### 2.5. Flow Cytometry

Cells were incubated with anti-CD16/32 (2.4G2) (BD Bioscience, San Jose, CA, USA) to block FcγR II/III, and then stained with various conjugated antibodies as indicated. A BD Cytofix/Cytoperm kit (BD Bioscience, San Jose, CA, USA) was used for intracellular cytokine and Foxp3 staining. BrdU incorporation was assayed using a BrdU Flow kit per manufacturer’s instructions (BD Bioscience, San Jose, CA, USA). Apoptosis was evaluated with an Annexin V-APC Flow kit (BD Bioscience, San Jose, CA, USA) following the manufacturer’s instructions. Stained cells were analyzed by FACSVerse or LSR II with FACSDiva (BD Bioscience, San Jose, CA, USA) or FCS Express (De Novo Software, Los Angeles, CA, USA) software.

### 2.6. Cytokine Assay

Cytokines in the culture supernatants and sera were measured using ELISA as we previously reported [30,31]. IL-4 and IFN-γ were measured with OptEIA kits (BD Bioscience, San Jose, CA, USA); IL-17, IL-17F, and IL-21 were measured with DuoSet ELISA kits (R&D Systems, Minneapolis, MN, USA). ELISA plates were developed with 3,3′,5,5′-tetramethylbenzidine (TMB) substrate (BD Bioscience, San Jose, CA, USA) and read with a microplate reader.

### 2.7. In Vivo Cytokine Capture Assay (IVCCA)

The in vivo cytokine levels for IL-4, IL-17, and IFN-γ were detected using an in vivo cytokine capture assay (IVCCA) as previously reported [10,32]. Mice were injected with biotinylated neutralizing monoclonal antibodies to capture the corresponding cytokines and bled 2–4 h later. IVCCA facilitates the measurement of cytokines in serum by increasing their in vivo half-lives. As such, the sensitivity of the in vivo cytokine assays increases by at least 30- to 1000-fold. Sera containing the complexes of cytokine/biotinylated anti-cytokine were assayed by ELISA.

### 2.8. Real-Time PCR

Total RNA was extracted from liver tissues with the RNeasy Mini Kit (Qiagen, Valencia, CA, USA), and cDNA was prepared using the High-Capacity cDNA Reverse Transcription Kit (Applied Biosystems, Foster City, CA, USA). Quantitative PCR (qPCR)was performed with the Platinum SYBR Green qPCR SuperMix-UDG w/RO on a Light Cycler 480 II (Roche, Indianapolis, IN, USA). The data were normalized to the 18S reference. Primers for *Col-I*, *Col-III* and *TGF-β1* were designed with OLIG 4.0 software (Molecular Biology Insights, Inc., Cascade, CO, USA) [29]. 

### 2.9. Statistical Analysis

All experimental data were analyzed and compared for statistically significant differences using the two-tailed Student’s *t* or Mann–Whitney *U* tests. ANOVA was used for comparing 3 or more groups. A *p*-value of < 0.05 was considered significant.

## 3. Results

### 3.1. RhoA-ROCK Inhibitor Fasudil Suppresses Hepatic Granuloma Formation and Fibrosis in Mice Infected with S. japonicum

**Figure 1:**
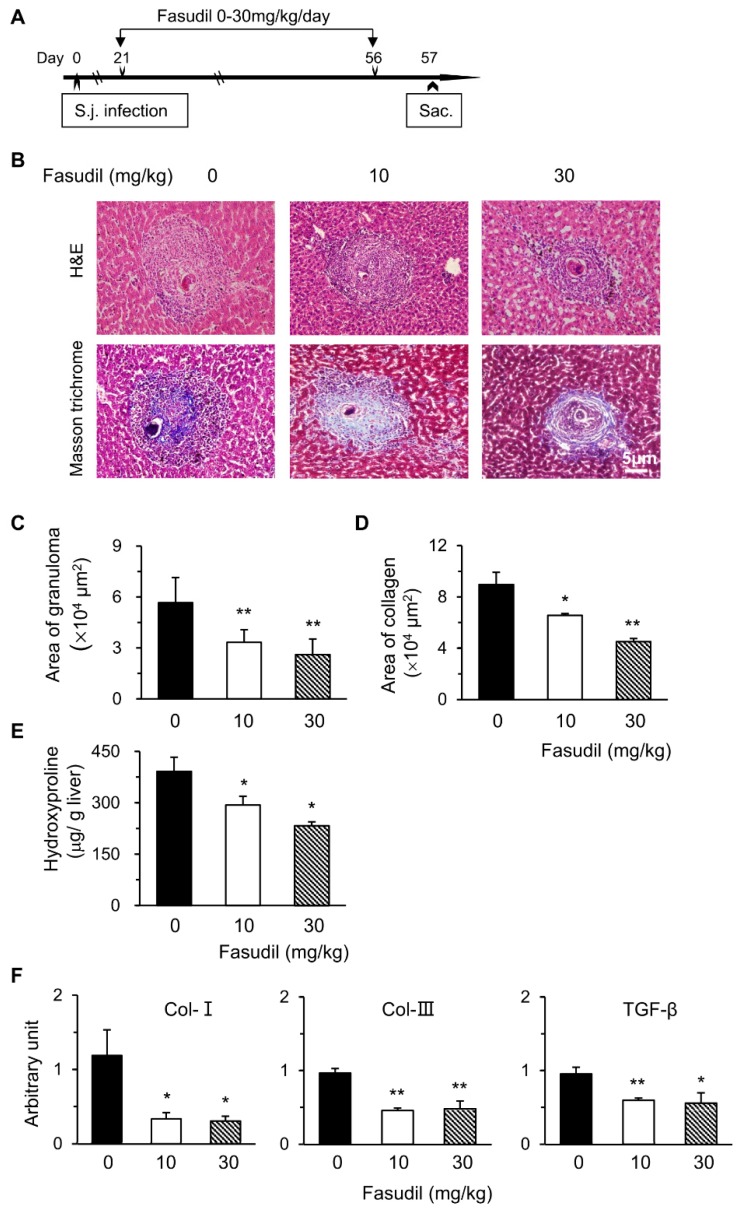
Fasudil therapy suppresses hepatic granuloma formation and fibrosis in mice infected with *Schistosoma japonicum*. (**A**) Female C57BL/6 mice were infected with *S. japonicum* (S.j.) and injected intraperitoneally (i.p.) with fasudil at 0, 10, and 30 mg/kg body weight daily for 5 weeks (day 21–56). Mice were sacrificed 24 h after the last injection. (**B**) Representative hematoxylin and eosin (H&E) and Masson trichrome staining are shown in liver sections. The sizes of (**C**) granulomas and (**D**) collagen areas by Masson trichrome staining around a single egg were measured. (**E**) Hepatic hydroxyproline levels were assayed. (**F**) The mRNA levels of *collagen type I* (*Col-I*), *Col-III*, and *transforming growth factor-1* (TGF-β1) in liver tissues were determined using real-time PCR. Data are normalized to an 18S reference and expressed as arbitrary units. Results are representative of three independent experiments (**C**–**F**, means + standard error (SE), *n* = 5–8). * *p* < 0.05, ** *p* < 0.01 vs. PBS-treated (fasudil 0) groups.

Schistosomiasis is an immunopathogenic disorder in which CD4^+^ T cells play key roles. Fasudil, a selective inhibitor for ROCK, plays multiple roles with these T cells [24,27]. Hence, we examined the potential effects of the RhoA-ROCK pathway in the pathogenesis of *S. japonicum* infection. We first investigated the effects of fasudil on hepatic granuloma formation and subsequent fibrosis. C57BL/6 mice were injected i.p. with fasudil (0–30 mg/kg body weight) daily starting at three weeks until eight weeks post-infection for a total five-week treatment (Figure 1A). Mice were sacrificed 24 h after the last injection to evaluate hepatic granuloma and fibrosis (Figure 1B–E). The sizes of the granuloma around a single egg were measured in liver sections with H&E staining (Figure 1B). Fasudil-treated mice showed much smaller granulomas than control mice (Figure 1C). The fibrotic areas around the granuloma were significantly reduced in fasudil-treated groups with both dosages as confirmed by Masson trichrome staining (Figure 1B,D). Consistent with these findings, hepatic hydroxyproline, a signature amino acid for fibrillar collagens comprising approximately 13.5% of the protein [33], also significantly decreased in fasudil-treated groups (Figure 1E). 

The mRNA levels of fibrosis-related genes in livers were investigated with real-time PCR. Fasudil treatment significantly suppressed the expression of *Col-I*, *Col-III*, and *transforming growth factor-1* (*TGF-β1*) (Figure 1F). Col-I and Col-III are the indicators of fibrosis. TGF-β1 can promote collagen synthesis and regulate the expression of several matrix metalloproteinases [34]. Hence, fasudil was able to downregulate the mRNA levels of hepatic collagen and *TGF-β1*. Collectively, our data suggest that fasudil therapy can suppress hepatic granuloma formation and subsequent fibrosis induced by *S. japonicum* infection. 

Fasudil therapy seems not to affect the worm burden in *S. japonicum* infection. To evaluate the roles of fasudil in the worm burden and fecundity of schistosomiasis, mice were injected i.p. with fasudil (0–30 mg/kg) daily starting one day prior to infection until eight weeks post-infection. Control groups were injected i.p. with PBS. Mice were sacrificed 24 h after the last injection (Figure S1A). We found that fasudil did not significantly affect the worm burden (Figure S1B). Fasudil treatment seemingly did not alter the fecundity of *S. japonicum*. Eggs deposited in the livers (Figure S1C) and in the feces (Figure S1D) did not differ markedly between fasudil-treated and control groups. These findings suggest that fasudil therapy does not affect worm burden and fecundity during *S. japonicum* infection.

### 3.2. Fasudil Inhibits Activation and Proliferation of CD4^+^ T Cells

**Figure 2:**
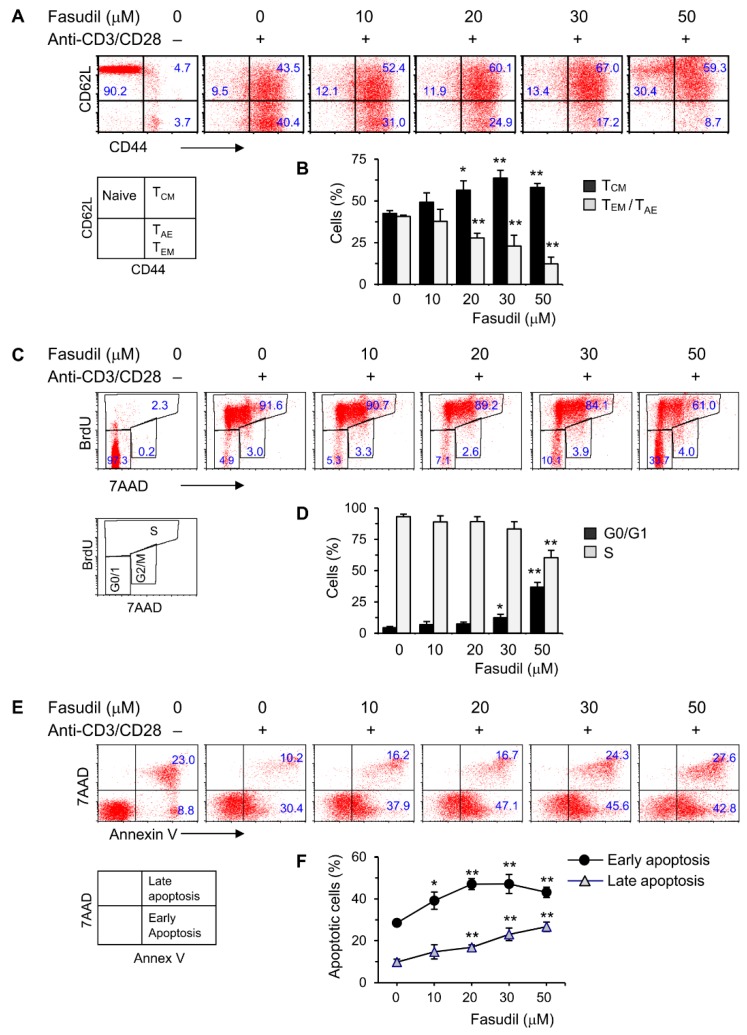
Fasudil inhibits T cell activation and proliferation and induces apoptosis in vitro. Naïve CD4^+^ T cells pooled from six mice were stimulated with plate-bound anti-CD3 plus free anti-CD28 for two days without or with fasudil (0–50 µM). Cells were collected and processed for FACS staining. (**A**,**B**) Cells were stained for CD44 and CD62L. (**A**) Representative dot plots are shown. (**B**) The percentages of central memory (T_CM_, CD44^hi^CD62L^hi^), activated effector (T_AE_) or effector memory (T_EM_) (both CD44^hi^CD62L^lo^) CD4^+^ T cells are summarized in a bar graph. (**C**,**D**) Cells were stained for 7AAD and BrdU. (**C**) Representative dot plots are shown. (**D**) The percentages of G0/G1 (BrdU^−^/7AAD^−^) and S phase (BrdU^+^) cells are summarized in a bar graph. (**E**,**F**) Cells were stained for 7AAD/Annex V. (**E**) Representative dot plots are shown. (**F**) The percentages of early (Annex V^+^/7AAD^−^) as well as late (Annex V^+^/7AAD^+^) apoptotic cells are summarized in a linear graph. Results (mean + SD of triplicates) are representative of three independent experiments. * *p* < 0.05, ** *p* < 0.01 vs. PBS-treated (fasudil 0) groups.

CD4^+^ T cells play an important role in the pathogenesis of schistosomiasis. To begin to understand the underlying fasudil mechanisms in the regulation of granuloma formation and fibrosis, we investigated the effects of fasudil on the activation, proliferation, and apoptosis of CD4^+^ T cells in vitro. To do so, purified naive CD4^+^ T cells were cultured with anti-CD3/CD28 for two days in the presence of different dosages of fasudil (0–50 µM). The activation and memory status of CD4^+^ T cells were assayed by fluorescence-activated cell sorting (FACS) staining with CD44/CD62L mAbs. The activated effector (T_AE_) or effector memory (T_EM_) CD4^+^ T cells (both CD44^hi^CD62L^lo^), which represent a pool of terminally differentiated cells and display immediate effector functions [35,36], were downregulated significantly by fasudil treatment. Naïve (CD44^lo^CD62L^hi^) and central memory (T_CM_, CD44^hi^CD62L^hi^) T cells increased accordingly with the treatment (Figure 2A,B). CD4^+^ T cell proliferation was evaluated using BrdU incorporation in vitro. Resting G0/G1 cells (BrdU^−^/7AAD^−^) increased, whereas S phase cells (BrdU^+^) decreased upon fasudil treatment at high concentrations (30–50 µM) (Figure 2C,D). The activation-induced cell death was determined by 7AAD/Annex V staining. Both early (Annex V^+^/7AAD^−^) as well as late (Annex V^+^/7AAD^+^) apoptotic cells significantly increased upon fasudil treatment in a dose-dependent manner (Figure 2E,F). Together, our data indicate that fasudil inhibits the activation and proliferation CD4^+^ T cells and induces the apoptosis of these cells in vitro.

### 3.3. Fasudil Impairs Th17 Differentiation and Cytokine Secretion In Vitro

**Figure 3:**
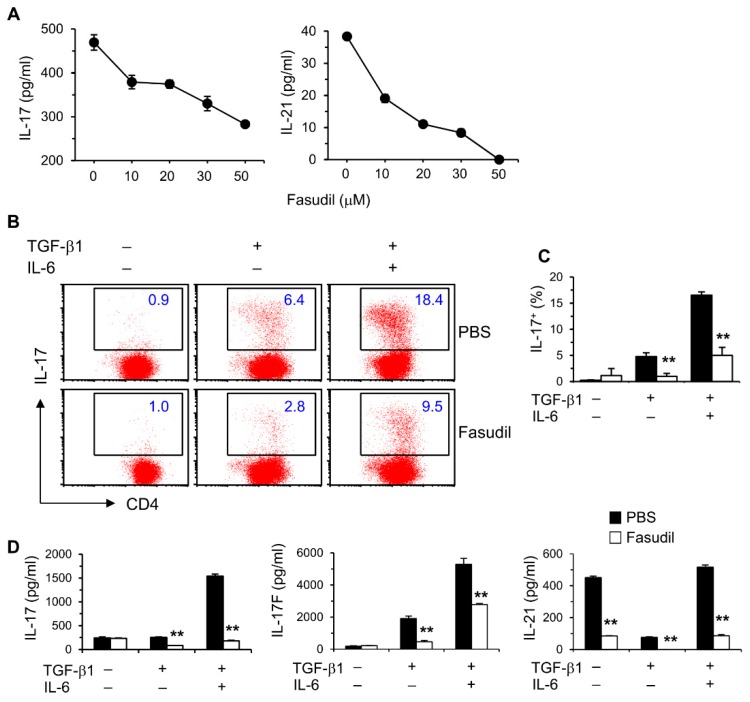
Fasudil blocks Th17 differentiation. (**A**) Naïve CD4^+^ T cells pooled from 8 mice were stimulated with anti-CD3/CD28 for two days without or with fasudil (0–50 µM). Interleukin (IL)-17 and IL-21 in the culture supernatants were determined by ELISA. (**B**,**D**) Naïve CD4^+^ T cells were differentiated under Th17/Treg conditions for four days and restimulated with PMA plus ionomycin for five hours in the presence of vehicle (PBS) or fasudil (30 µM) throughout the culture. Cells were collected for IL-17 intracellular staining. Percentages of IL-17^+^ CD4^+^ T cells are shown (**B**) in representative dot plots and (**C**) summarized in a bar graph. (**D**) IL-17, IL-17F, and IL-21 in the culture supernatants were determined by ELISA. Data (mean + SD of triplicates) are representative of two independent experiments. ** *p* < 0.01 compared with PBS-treated groups.

We reported that IL-17 levels in vivo are elevated during the whole course of *S. japonicum* infection [10]. We therefore sought to determine whether blocking the RhoA-ROCK pathway would influence Th17 differentiation and effector cytokine secretion in vitro. Purified naïve splenic CD4^+^ T cells from C57BL/6 mice were cultured under non-polarizing conditions with anti-CD3/CD28 stimulation for two days; fasudil effectively blocked the production of Th17 effector cytokines, IL-17 and IL-21, in a dose-dependent manner (Figure 3A). Under Th17 skewing conditions, IL-17-secreting cells were significantly reduced in fasudil-treated CD4^+^ T cells determined per FACS analysis (Figure 3B,C). Consistently, IL-17 levels in the culture supernatants were markedly reduced; IL-17F and IL-21 were also downregulated in the cultures treated with fasudil as analyzed by ELISA (Figure 3D). Notably, IL-21 has an autocrine effect in promoting Th17 maturation [37]. These data demonstrate that fasudil significantly inhibits Th17 differentiation and effector cytokine secretion.

### 3.4. Fasudil Upregulates iTreg Cells

**Figure 4:**
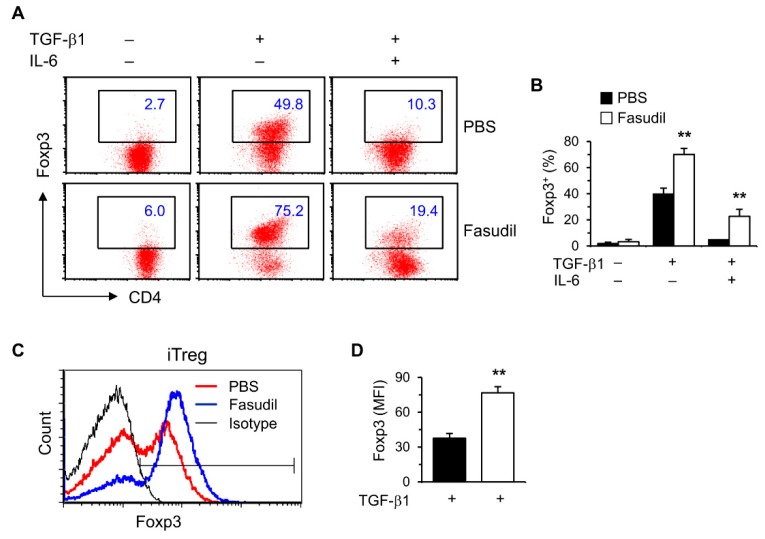
Fasudil upregulates iTreg cells. Naïve CD4^+^ T cells were differentiated under Th17/Treg conditions for 4 days and restimulated with PMA plus ionomycin for 5 h in the presence of vehicle (PBS) or fasudil (30 µM) throughout the culture. Cells were collected for Foxp3 intracellular staining. Percentages of Foxp3^+^ CD4^+^ T cells are shown in (**A**) representative dot plots and (**B**) summarized in a bar graph. (**C**) Under iTreg conditions with TGF-β1, the mean fluorescence intensity (MFI) of Foxp3 is shown in a histogram gated on CD4^+^ T cells. (**D**) The MFI of PBS versus fasudil-treated groups is summarized in a bar graph. Data (mean + SD of triplicates) are representative of two independent experiments. ** *p* < 0.01 compared with PBS-treated groups.

Treg cells play important roles in suppressing immunopathological injury in livers after schistosome infection. To observe the effects of fasudil on induced Treg (iTreg) cells, purified naïve splenic CD4^+^ T cells were cultured in vitro under Treg polarizing conditions with TGF-β1 without IL-6. Treg-specific transcription factor Foxp3 was significantly upregulated in FACS analysis (Figure 4). Both the percentages (Figure 4A,B) and mean fluorescence intensity (MFI) (Figure 4C,D) were markedly increased in fasudil-treated CD4^+^ T cells. 

### 3.5. Fasudil Therapy Downregulates Th2 and Th17 Cytokine Levels In Vivo in Infected Mice

**Figure 5:**
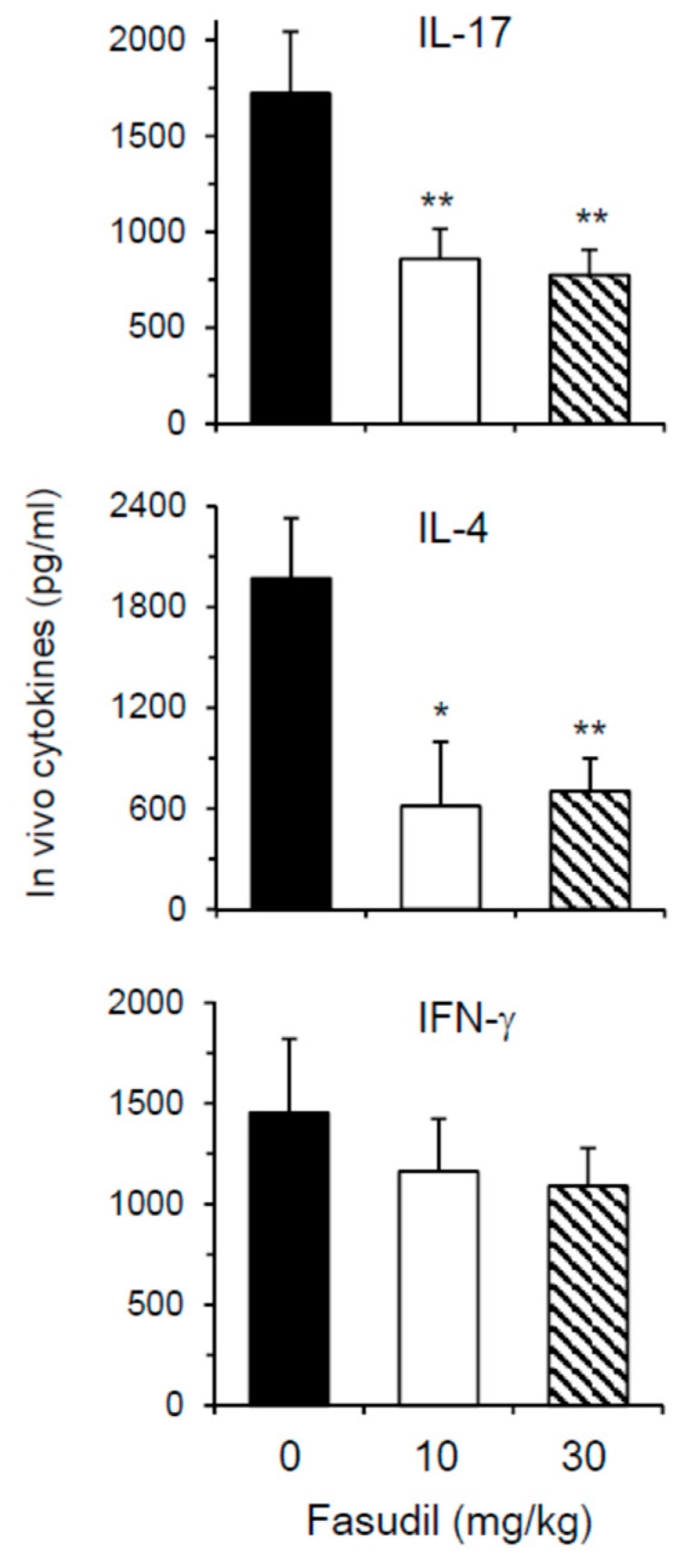
Fasudil reduces in vivo Th17 and Th2 cytokine post-infection. Mice were infected with *S. japonicum* and injected i.p. with fasudil at 0, 10 and 30 mg/kg for 5-weeks (d 21–d 56). The levels of IL-17, IL-4, and IFN-γ in the peripheral blood were assayed at 8 weeks post-infection by IVCCA. Results (*n* = 5–8 per group) are representative of two independent experiments. * *p* < 0.05, ** *p* < 0.01 vs. PBS-injected mice.

Th2 and Th17 cells play key roles in the pathogenesis of schistosomiasis. We next wanted to determine whether fasudil therapy affects the effector cytokine secretion in vivo. As most cytokines are used, catabolized, or excreted shortly after they are produced in vivo, direct measurement of in vivo cytokine secretion is difficult [32]. IVCCA was established in our laboratory to determine the circulating levels of several cytokines [10]. In vivo IL-4 and IL-17, representative Th2 and Th17 cytokine, respectively, were markedly downregulated in mice injected i.p. with fasudil daily starting three weeks post-infection for five weeks (Figure 5); whereas, in vivo IFN-γ, representative Th1 cytokine, was comparable between fasudil- and PBS-injected mice. These data are consistent with the observed in vitro effects of fasudil on the effector cytokine secretions from Th2 and Th17 cells.

### 3.6. Fasudil Induces Apoptosis of Hepatic Stellate Cells

**Figure 6:**
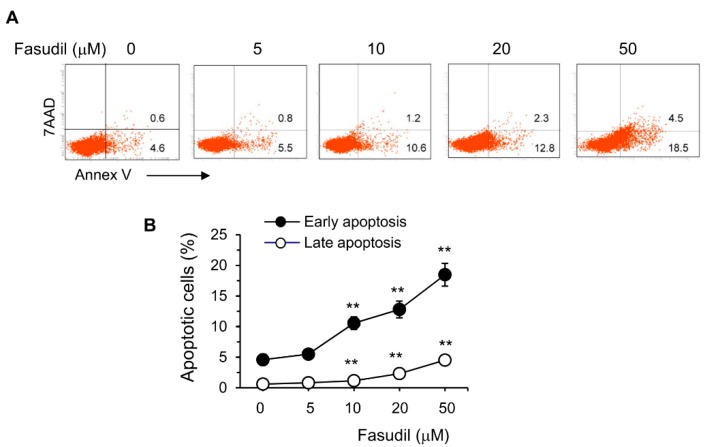
Fasudil promotes apoptosis of hepatic stellate cells. HSC-T6 cells were cultured for 2 days without or with fasudil (0–50 µM). Cells were collected and processed for 7AAD/Annex V staining. (**A**) Representative dot plots and (**B**) the percentages of early (Annex V^+^/7AAD^−^) and late (Annex V^+^/7AAD^+^) apoptotic cells are summarized in a linear graph. Results (mean + SD of triplicates) are representative of two independent experiments. ** *p* < 0.01 vs. PBS-treated groups.

The HSC is the main cell type responsible for collagen deposition and fibrosis formation in the liver [5]. To observe the effect of fasudil on the induction of apoptosis of HSC, HSC-T6 cells were cultured in vitro with different concentrations of fasudil. We found that fasudil was able to induce the apoptosis of HSCs in a dose-dependent manner, suggesting its direct apoptotic role with this key fibrogenic cell (Figure 6).

## 4. Discussion

Fasudil, a selective inhibitor for the RhoA-ROCK pathway, has been used in clinic as a vasodilator [18,19,20] and for RhoA-mediated functional studies [21,22,23]. We previously reported that fasudil can partially block Th2 differentiation and OVA-induced allergic airway inflammation [27]. However, the roles of fasudil for Th17 cells and in the pathogenesis of *S. japonicum* infection remain unclear. 

The main pathogenesis for schistosomiasis is the granuloma formation around deposited eggs and sequent fibroses in the liver and other affected tissues [2,5]. Persistent fibrosis in chronic infection may cause hepatic cirrhosis with high mortality [9]. In this study, we found that fasudil therapy could inhibit hepatic fibrosis induced by *S. japonicum* infection. Through long-term (five weeks) fasudil injection started shortly before the egg deposition occurred, i.e., 21 days post-infection, granuloma size and fibrotic area were significantly reduced. Consistently, the hepatic hydroxyproline content, the main constitute of collagen, was markedly reduced. 

Hepatic fibrosis is mainly caused by liver-injury-mediated inflammation, in which HSCs play critical roles upon their activation by inflammatory cytokines and mediators [5,11]. HSCs are one of the main effector cells in hepatic fibrosis, partly due to their capacity to transdifferentiate into collagen-producing myofibroblast [38]. The latter secretes a large amount of collagen and syntheses the extracellular matrix, distorts normal tissue structure, and eventually leads to hepatic fibrosis [7,38,39]. HSCs also orchestrate the persistent synthesis and secretion of profibrotic cytokines and tissue-destructive enzymes such as matrix metalloproteinases [40]. We found that fasudil can directly induce the apoptosis of HSCs in a dose-dependent manner (Figure 6). Fasudil therapy can downregulate the mRNA expression of fibrogenic molecules in the liver, such as Col-I, Col-III, and TGF-β1 (Figure 1F). However, the effects of fasudil on the matrix metalloproteinase pathway remain elusive. Further studies are needed to investigate the related molecules and pathways involved. 

Considerable evidence has demonstrated that Th17 and Treg cells are key players in the pathogenesis of schistosomiasis. The severity of hepatic pathogenesis is correlated with IL-17 activity [7,8,9,10]. By measuring in vivo IL-17 level during infection, we found that Th17 cells are actively involved in early anti-infection immunity and late immunopathogenesis for granuloma and fibrosis in schistosomiasis [10]. Here, our results indicated that fasudil blocks Th17-differentiatin in vitro and suppresses the in vivo circulating IL-17 levels, which may be related to its anti-pathogenesis effects for schistosomiasis. Treg cells play a pivotal role in limiting hepatic immunopathological damage in schistosomiasis [41]. We found that fasudil was able to markedly upregulate iTreg cells, which might also contribute its anti-fibrotic roles. Fasudil can inhibit both ROCK1 and ROCK2 isoforms. Whether these two isoforms have distinct roles in the development of hepatic fibrosis remains mostly unknown. Our current findings are based on murine cells and models. As significant differences exist between mouse and human immune systems, one of our future studies will be to explore the roles of fasudil on human hepatic fibrosis in schistosomiasis.

Fasudil therapy may be also beneficial for hepatic fibrosis caused by diabetes, chronic hepatitis, toxic agents, injuries, etc. [22,42,43]. As ROCK is expressed in hepatic tissues in hepatocellular carcinoma (HCC) and suppresses the cell cycle and the p53 or NF-κB-mediated apoptosis pathway in HCC, fasudil may be also a beneficial approach to HCC therapy [44,45]. These observations demonstrate the broad potential prospects for fasudil therapy. 

In summary, we demonstrated that fasudil regulates hepatic granuloma formation and fibrosis due to *S. japonicum* infection. These effects of fasudil may be related to the downregulation of CD4^+^ T cell activation and Th17 differentiation, and upregulation of Treg cells. Fasudil may have direct anti-fibrotic effects, such as inducing apoptosis to hepatic stellate cells and suppressing the expression of fibrotic genes. More in-depth investigations are needed to fully characterize the cellular and molecular mechanisms through which fasudil regulates the pathogenesis of schistosomiasis before it can be developed into a novel anti-fibrotic therapy for schistosomiasis and perhaps other hepatic disorders. We have highlighted our main findings in the Appendix A.

## Supplementary Materials

The following are available online at https://www.mdpi.com/2073-4409/8/10/1262/s1, Figure S1: Fasudil does not affect worm burden by *S. japonicum* infection.

## Abbreviations

Col-I	collagen type I
ELISA	enzyme-linked immunosorbent assay
EPG	eggs per gram
FACS	fluorescence-activated cell sorting
GDP	guanosine diphosphate
GTP	guanosine triphosphate
HCC	hepatocellular carcinoma
H&E	hematoxylin and eosin
HSCs	hepatic stellate cells
IL	interleukin
i.p.	intraperitoneally
IVCCA	in vivo cytokine capture assay
KO	knock-out
PBS	phosphate buffer saline
PMA	phorbol 12-myristate 13-acetate
RhoA	ras homolog family member A
ROCK	Rho-associated kinase
*S. japonicum*	*Schistosoma japonicum*
Stat3	signal transducer and activator of transcription 3
Th	T helper
TGF-β1	transforming growth factor-β1
Treg	regulatory T
WT	wild-type

## Appendix A

RhoA-ROCK inhibitor fasudil suppresses Th17 differentiation and cytokine secretion in vitro.

Fasudil upregulates iTreg cells.

Fasudil induces the apoptosis of hepatic stellate cells and downregulates the expression of hepatic fibrogenic genes, Col-I, Col-III, and TGF-β1.

Fasudil therapy inhibits hepatic granuloma formation and fibrosis by *Schistosoma japonicum* infection.
