# Supplementary material for: Inhibition of Rho-Kinase Downregulates Th17 Cells and Ameliorates Hepatic Fibrosis by Schistosoma japonicum Infection
Source: Cells. 2019 Oct 16;8(10):1262. doi: 10.3390/cells8101262 (PMC6829618; doi:10.3390/cells8101262)
Supplement: Supplementary file 1 [file cells-08-01262-s001.pdf]

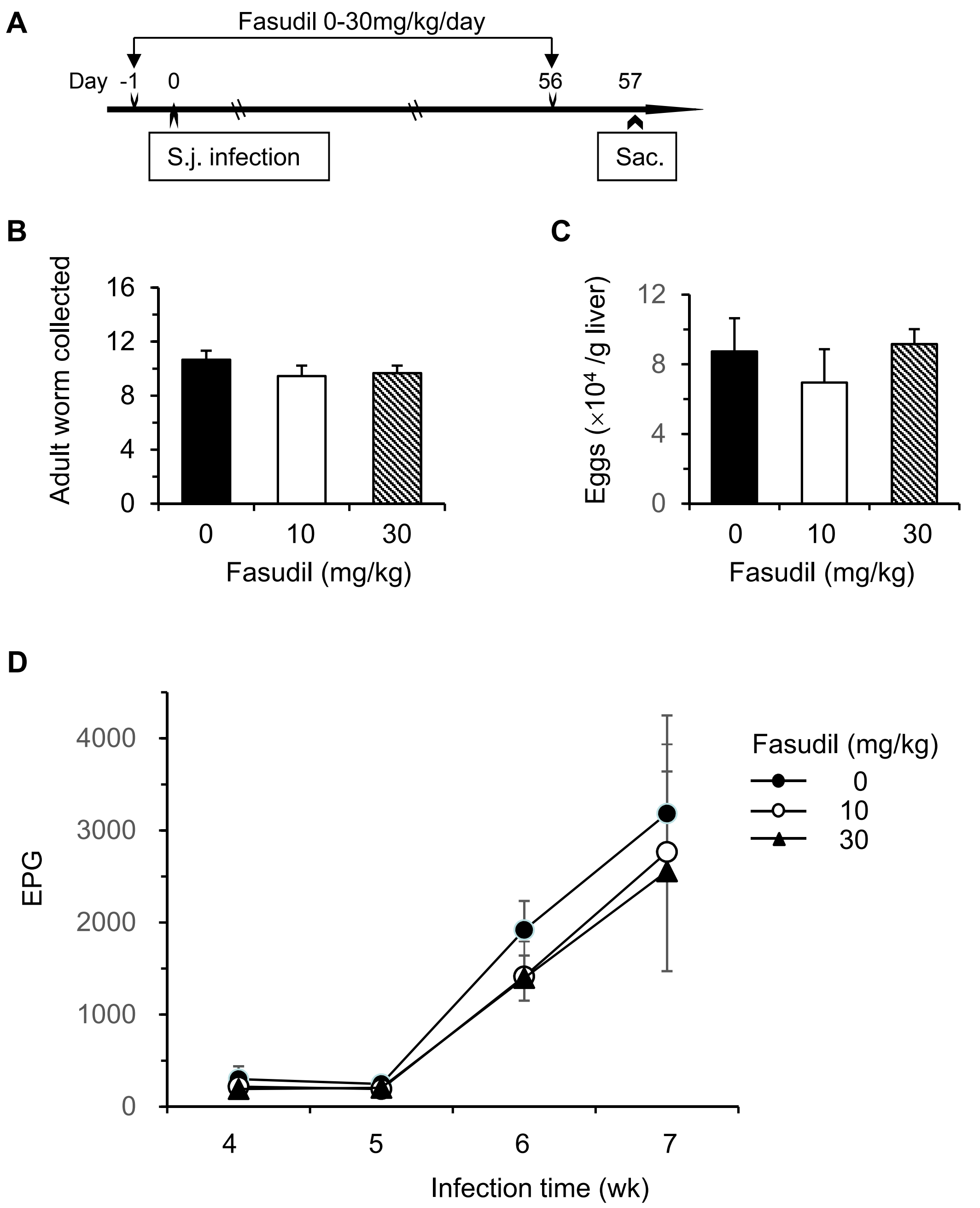

Figure S1. Fasudil does not affect worm burden by *S. japonicum* infection.

Mice were infected with *S. japonicum* and injected i.p. with fasudil at 0–30 mg/kg for 8 weeks (d -1~d 56). Mice were sacrificed 24 h after the last injection for worm burden assays (**A**). Adult worms collected from portal and mesenteric veins per mouse (**B**), eggs in livers (**C**) and eggs per gram (EPG) of feces (**D**) are shown. Results are representative of three independent experiments.
